# Supporting data for characterization of the busulfan metabolite EdAG and the Glutaredoxins that it adducts

**DOI:** 10.1016/j.dib.2015.09.002

**Published:** 2015-09-10

**Authors:** Michele Scian, William M. Atkins

**Affiliations:** Department of Medicinal Chemistry, University of Washington, United States

## Abstract

This article describes data related to a research article titled “The Busulfan Metabolite EdAG Irreversibly Glutathionylates Glutaredoxins” [1]. EdAG is an electrophilic GSH analog formed in vivo from busulfan, which is used in hematopoietic stem cell transplants. EdAG glutathionylates Glutaredoxins (Grx's) but not glutathione transferase A1-1 (GSTA1-1) in vitro. This article includes a complete NMR characterization of synthetic EdAG including homonuclear and heteronuclear correlation spectra. Also included are mass spectra of peptides from Grx's or GSTA1-1 that have cys residues that do not react with EdAG.

**Specifications Table**

**Table:** 

Subject area	*Chemistry*
More specific subject area	*Protein chemistry and drug metabolism*
Type of data	*1 Table, multiple spectra*
How data was acquired	*NMR with* Agilent DD2 500 MHz instrument*, mass spectrometry with Waters Synapt G2-S Q-TOF.*
Data format	*Standard NMR or mass spec format*
Experimental factors	*Standard sample handling*
Experimental features	*All NMR spectra were recorded in 90:10 D2O:H2O at ∼5* *mM EdAG, pH 3. All mass spectra were at 50 micromolar protein pH 7.4 unless otherwise noted in figures.*
Data source location	*Seattle, WA USA*
Data accessibility	*Data are accessible in this article only*

**Value of the data**

• EdAG is a potentially important metabolite of the therapeutic agent busulfan, but EdAG is not commercially available, and not completely characterized in the literature.
• Future studies concerning effects of EdAG in biological systems would require its synthesis and characterization, which will be facilitated by the NMR data included here.
• Mass spectra of tryptic peptides of Grx's and GSTA1-1 that are not adducted by EdAG will be valuable benchmarks for future work aimed to determine the extent of EdAG reaction in vivo.

## 1 Data

EdAG has been shown to irreversibly glutathionylate and inhibit Grx's which play a critical role in the glutathionylation and deglutathionylation of many proteins. Grx's are important for many cellular regulatory processes [1]. Many other redoxins that contain active site cys residues in GSH binding sites, or other proteins with nucleophilic cys residues, may be targets for EdAG as well. Collectively, these reactions could contribute to the clearance, distribution, or toxicity of busulfan. Further studies on the mechanism of busulfan and its metabolite EdAG are required. However, EdAG is not commercially available and requires synthesis from GSH. The NMR characterization reported here will facilitate future efforts to synthesize EdAG. In addition, Reference [1] documents the relative specificity of EdAG for cys residues in GSH binding sites, and the mass spectral data included here demonstrate the lack of reaction of EdAG at other cys residues and they demonstrate the apparent oxidation of Grx's, independent from EdAG treatment.

A scheme depicting the overall two step synthesis is shown in the Figs. 1 and 2 shows the homo-and heteronuclear correlations characterized by NMR. Figs. 3–7 are 2D-homo- and heteronuclear correlation spectra as indicated (Figs. 8–12).

## 2 Experimental design, materials and methods

**Fig. 1:**
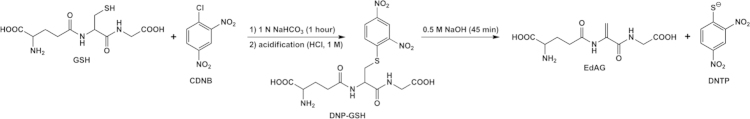
Synthesis of EdAG.

The synthetic product from Fig. 1 was fully characterized by ^1^H, ^13^C and ^1^H–^13^C NMR. ^1^H NMR (D_2_O, pH*∼*3): *δ* 2.17 (q, *J*=7.3 Hz, 2H), 2.58 (td, *J*=7.3, 4.5 Hz, 2H), 3.82 (t, *J*=6.4 Hz, 1H), 4.00 (s, 2H), 5.68 ppm (s, 1H), 5.74 ppm (s, 1H). ESI-MS (positive ion mode, [MH]^+^=274.1 m/z). The yield of EdAG from starting S-(2,4-dinitrophenyl)glutathione was 60% (Table 1).

### 2.1 NMR spectroscopy

All NMR experiments were performed at 25 °C on a 499.73 MHz Agilent DD2 spectrometer equipped with either a 5 mm triple-resonance ^1^H(^13^C/^15^N) or a 5 mm AutoX Dual Broadband, *z*-axis pulsed-field gradient probe head.

For characterization and spectral assignment purposes, the EdAG samples were *∼*5 mM solutions in either unbuffered D_2_O (99.9% D, Cambridge Isotopes) or H_2_O/D_2_O 90:10 at pH*∼*3. For ^13^C NMR spectral acquisition the sample concentration was*∼*40 mM in unbuffered H_2_O/D_2_O 90:10 at pH*∼*3.

The 2,2-dimethyl-2-silapentane-5-sulfonate sodium salt (DSS) was used as the internal chemical shift reference and set to 0.0 ppm under all conditions. Proton spectra were acquired at a resolution of 16 k complex points in the time domain with 64 accumulations each (sw=5000 Hz, *d*1=1s) and WATERGATE [2] or WET [3,4] solvent suppression whenever required. The ^13^C spectrum was acquired at a resolution of 8 k complex points in the time domain with 10,000 accumulations (*sw*=28,000 Hz, *d*1=3s).

EdAG proton resonances were assigned through a combination of two-dimensional (2D) DQF-COSY [5], TOCSY) [6] and NOESY [7,8] experiments.

All homonuclear 2D experiments were acquired with 1024 complex data points in the t2 time domain (*sw*=5000 Hz, *d*1=1.5s) and 8 (for DQF-COSY and TOCSY) or 16 (for NOESY) scans were averaged for each of the 400 increments in the t1 domain.

The TOCSY spectrum was recorded with a 50 ms DIPSI [9] spin-lock sequence (*γ*B1/2*π* =6 kHz) and water suppression was achieved by a WATERGATE sequence applied prior to acquisition.

For the NOESY experiment a mixing time of 750 ms was employed and the solvent suppressed by transmitter presaturation during the relaxation (*d*1) and the mixing (mix) delay. A Stimulated Cross peak Under Bleached Alphas (SCUBA) [10] pulse sequence element with a delay of 50 ms at the end of the first presaturation period was used to recover the saturated Hα resonances.

EdAG carbon resonances were assigned through a combination of two dimensional ^1^H–^13^C HSQC [11] and ^1^H–^13^C HMBC [12], both acquired at natural isotopic abundance with 1024 complex data points in the t2 time domain (*sw*=5000 Hz, *d*1=3 s) and 128 averaged accumulations for each of the 200 increments in the t1 domain. The employed ^1^H–^13^C HSQC pulse sequence featured a sensitivity enhancement scheme and gradients for coherence selection and water suppression [13,14]. The spectral window in the indirect dimension was set at 160 ppm (20105.1 Hz) and centered at 75 ppm. The gradient-selected, absolute value ^1^H–^13^C HMBC featured a Shaka6 composite 180° pulse to achieve broadband inversion [15], a three-step low-pass J-filter [16,17] to suppress one-bond correlations (the high and low one-bond ^1^*J*_CH_ coupling constants were set to 160 and 110 Hz, respectively), and WET solvent suppression during the relaxation delay. Multiple-bond ^n^*J*_CH_ coupling constant was set to 7.5 Hz and the spectral window in the indirect dimension was set at 240 ppm (30154.5 Hz) and centered at 110 ppm.

All two-dimensional spectra were acquired in phase-sensitive Hypercomplex 2D mode with States-TPPI for quadrature detection in F1 [18,19].

The NMR data were analyzed using MNova 10.0 processing software (Mestrelab Research, Santiago de Compostela, Spain).

### 2.2 Mass Spectrometry

All mass analyses were performed on a SYNAPT G2-Si quadrupole time of flight spectrometer (Waters, Milford, MA). To ensure high mass accuracy throughout an analysis, a lock mass (leucine enkephalin, [M+H]^+^=556.2771 Da) was sampled every 60 s during the run.

For the intact protein analysis,*∼*5 μg were applied to a POROS-R1 column (150×2.1 mm^2^, 10 μm particle size, Applied Biosystem) and subjected to a binary mobile phase linear gradient (A=0.1% F.A.; B=ACN+0.1% F.A.) from 10% to 95% B over the course of 17 min, at a flow rate of 0.3 ml/min. The MS spectra acquisition was done in positive mode, scanning through a *m*/*z* range of 200–3000 Da.

For the tryptic digestion LC-MS/MS analysis,*∼*350 ng digested protein were resolved on an UPLC BEH C18 column (100×1.0 mm^2^, 1.7 μm particle size, Waters) and subjected to a linear gradient (A=0.1% F.A.; B=ACN+0.1% F.A.) from 5% to 50% B over the course of 24 min, at a flow rate of 0.08 ml/min.

The MS spectra were acquired with data dependent acquisition (DDA), with a survey scan of 1 s through a *m*/*z* range of 50–2000 Da, and a subsequent MS/MS scan from 50 to1200 Da for 1 s with the trap collision energy of 30 eV. The mass error was found, in all instances, to be under 5 ppm.

All data acquisition, processing and visualization were performed using MassLynx (Waters). Peptides were manually assigned using exact mass and MS/MS spectra with the aid of protein prospector (prospector.ucsf.edu).

## Figures and Tables

**Fig. 2:**
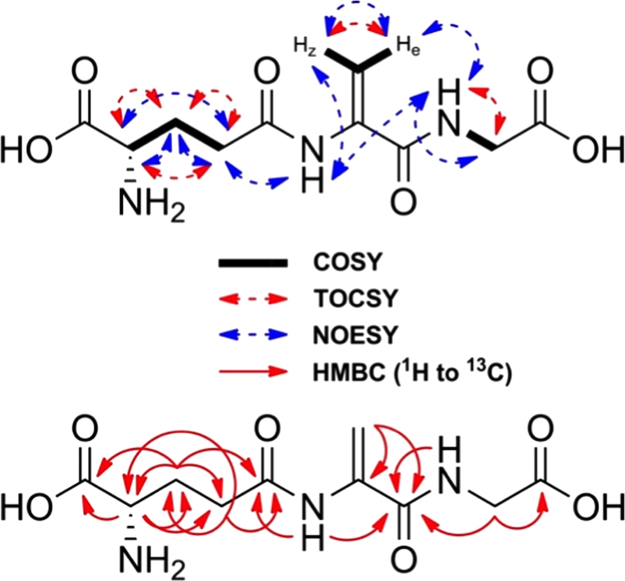
Schematic representation of the homo- and heteronuclear correlations observed in the NMR spectra a*∼*5 mM solution of EdAG in unbuffered H_2_O/D_2_O 90:10 at pH*∼*3.

**Fig. 3:**
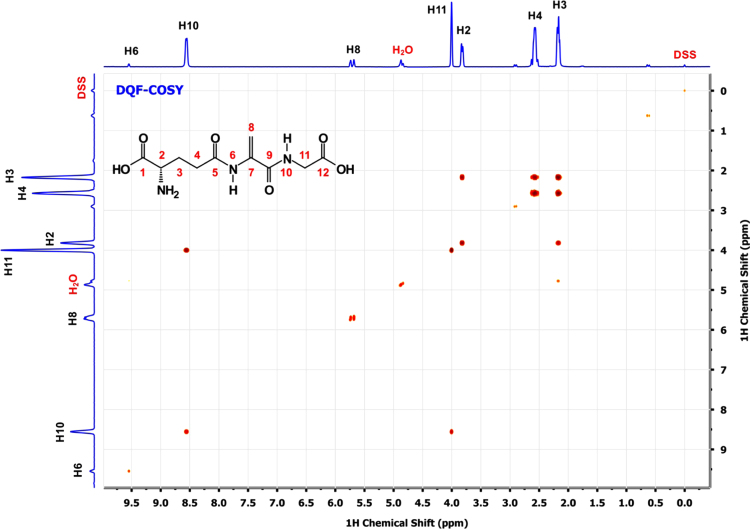
2D ^1^H–^1^H DQF-COSY spectrum of a*∼*5 mM solution of EdAG in unbuffered H_2_O/D_2_O 90:10 at pH*∼*3. 2-2-dimethyl-2-silapentane-5-sulfonate sodium salt (DSS) was used as the internal chemical shift reference.

**Fig. 4:**
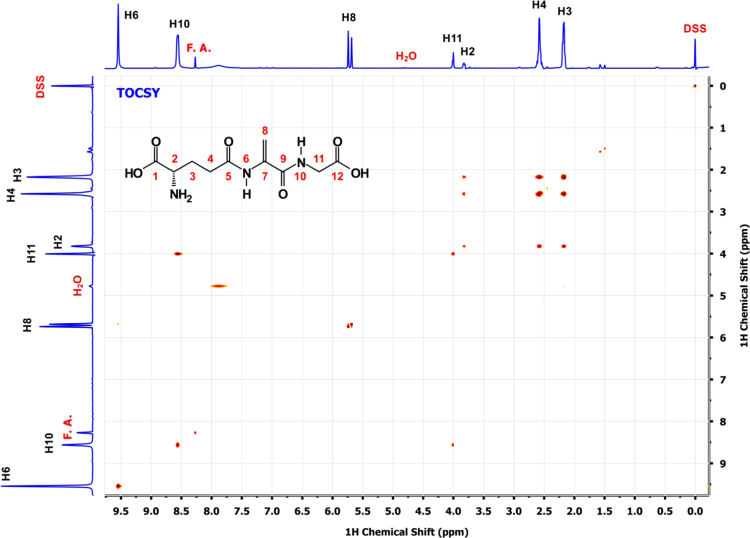
2D ^1^H–^1^H TOCSY spectrum of a*∼*5 mM solution of EdAG in unbuffered H_2_O/D_2_O 90:10 at pH*∼*3. 2-2-dimethyl-2-silapentane-5-sulfonate sodium salt (DSS) was used as the internal chemical shift reference.

**Fig. 5:**
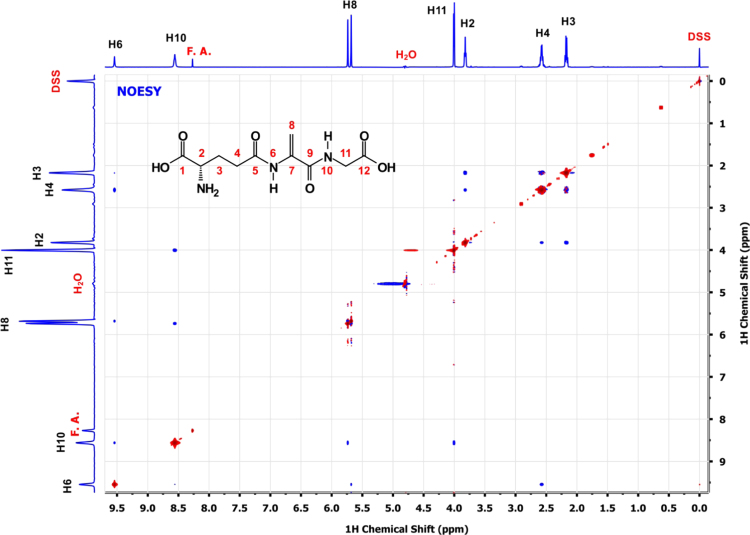
2D ^1^H–^1^H NOESY spectrum of a*∼*5 mM solution of EdAG in unbuffered H_2_O/D_2_O 90:10 at pH*∼*3. 2-2-dimethyl-2-silapentane-5-sulfonate sodium salt (DSS) was used as the internal chemical shift reference.

**Fig. 6:**
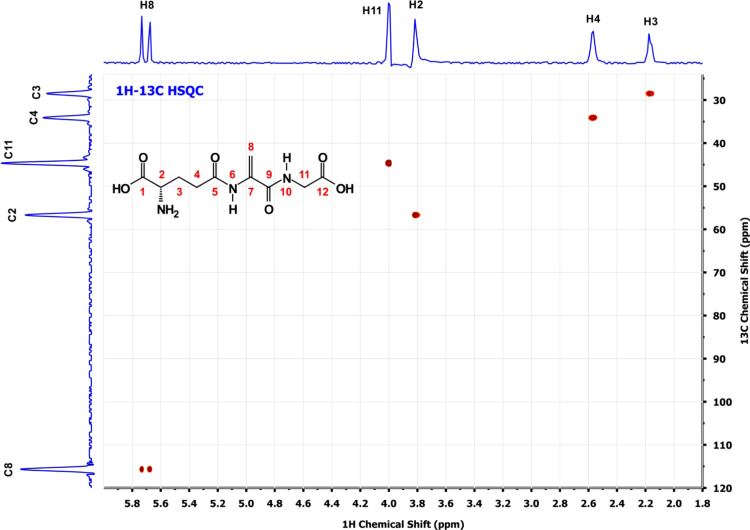
^1^H–^13^C HSQC spectrum of a*∼*5 mM solution of EdAG in unbuffered H_2_O/D_2_O 90:10 at pH*∼*3. 2-2-dimethyl-2-silapentane-5-sulfonate sodium salt (DSS) was used as the internal chemical shift reference.

**Fig. 7:**
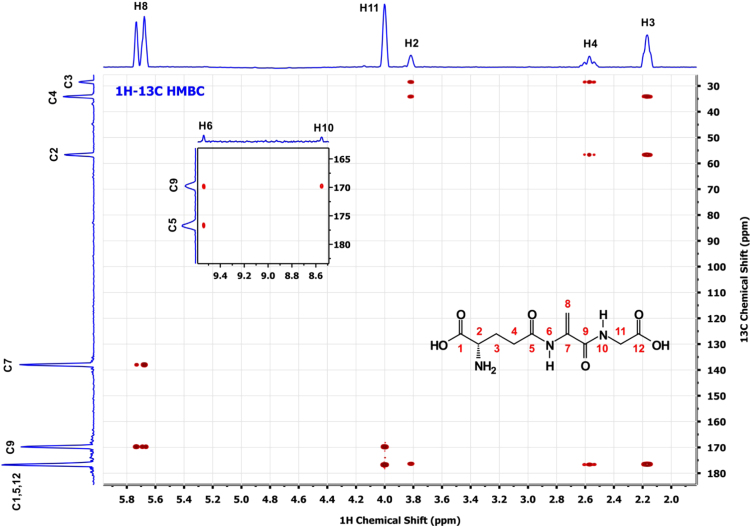
^1^H–^13^C HMBC spectrum of a*∼*5 mM solution of EdAG in unbuffered H_2_O/D_2_O 90:10 at pH*∼*3. 2-2-dimethyl-2-silapentane-5-sulfonate sodium salt (DSS) was used as the internal chemical shift reference.

**Fig. 8:**
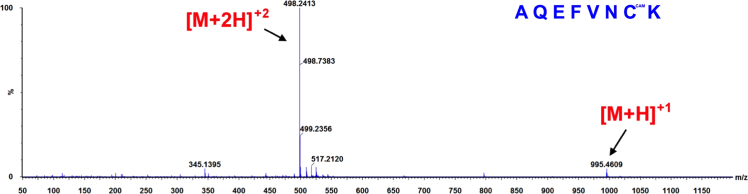
ESI-MS spectra of the hGrx-1 tryptic peptide AQEFVNCK obtained from a Grx-1/EdAG mixture (50 μM Grx-1+1 mM EdAG) incubated for 24 h at 37 °C in PBS, pH 7.4 and in presence of 250 μM TCEP. The observed molecular weight indicates that the peptide contains a carbamidomethyl derivative (CAM) of the cys residue as a result of reaction with iodoacetamide. The result was confirmed by high mass accuracy MS/MS data (not shown).

**Fig. 9:**
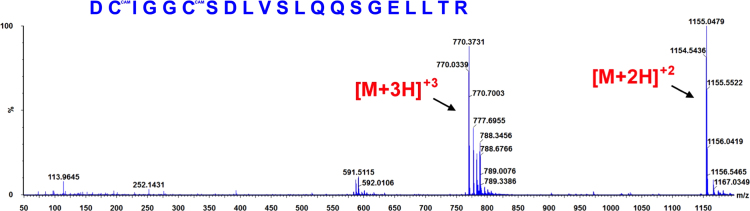
ESI-MS spectra of the hGrx-1 tryptic peptide DCIGGCSDLVSLQQSGELLTR obtained from a Grx-1/EdAG mixture (50 μM Grx-1+1 mM EdAG) incubated for 24 h at 37 °C in PBS, pH 7.4 and in presence of 250 μM TCEP. The observed molecular weight indicates that the peptide contains two carbamidomethyl derivatives (CAM) of the cys residues as a result of reaction with iodoacetamide. The result was confirmed by high mass accuracy MS/MS data (not shown).

**Fig. 10:**
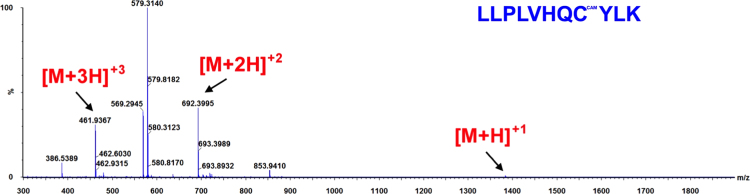
ESI-MS spectra of the hGrx-2(41–164) tryptic peptide LLPLVHQCYLK obtained from a Grx-2/EdAG mixture (50 μM Grx-1+1 mM EdAG) incubated for 24 h at 37 °C in PBS, pH 7.4 and in presence of 250 μM TCEP. The observed molecular weight indicates that the peptide contains a carbamidomethyl derivative (CAM) of the cys residue as a result of reaction with iodoacetamide. The result was confirmed by high mass accuracy MS/MS data (not shown).

**Fig. 11:**
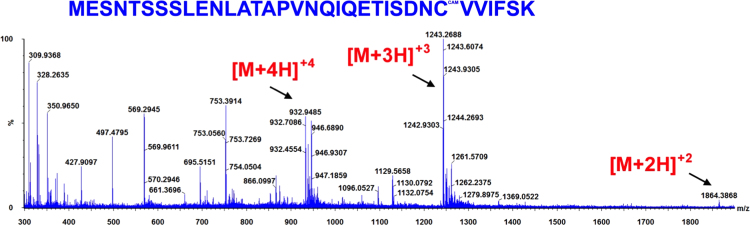
ESI-MS spectra of the hGrx-2(41–164) tryptic peptide MESNTSSSLENLATAPVNQIQETISDNCVVIFSK obtained from a Grx-2/EdAG mixture (50 μM Grx-1+1 mM EdAG) incubated for 24 h at 37 °C in PBS, pH 7.4 and in presence of 250 μM TCEP. The observed molecular weight indicates that the peptide contains a carbamidomethyl derivative (CAM) of the cys residue as a result of reaction with iodoacetamide. The result was confirmed by high mass accuracy MS/MS data (not shown).

**Fig. 12:**
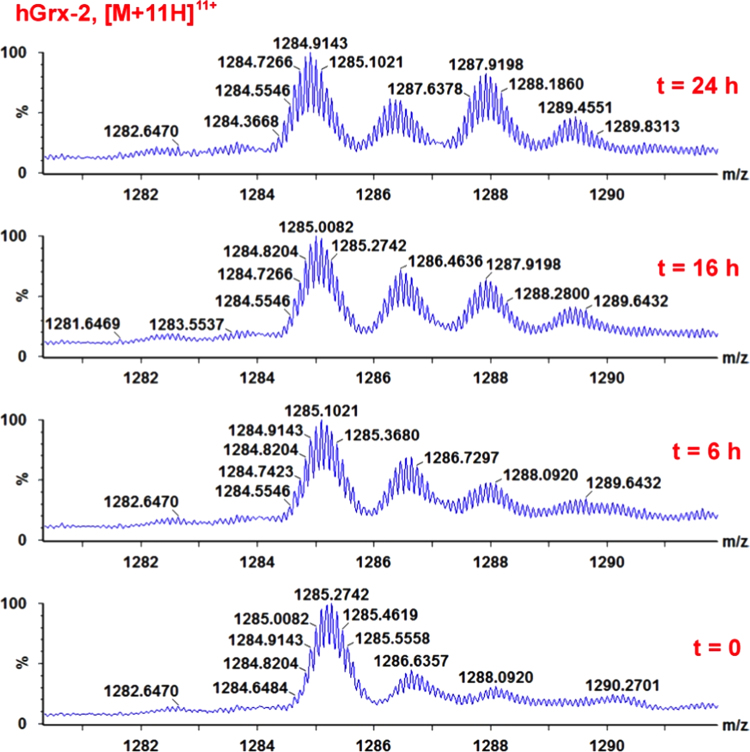
ESI-MS spectra of the reaction mixture obtained from 1 mM EdAG and 50 μM Grx−2 at 0, 6, 16 and 24 h. Only the [M+11H]^11+^ charge state of the intact an unmodified protein is shown to clearly illustrate the time-dependent oxidation of the four cysteine residues. Methionine oxidized species of the intact protein were also formed during the time of the incubation. The reaction was conducted at 37 °C in PBS, pH 7.4, and in presence of 250 μM TCEP.

**Table 1:** NMR Chemical Shift assignment of a*∼*5 mM solution of EdAG in unbuffered H_2_O/D_2_O 90:10 at pH*∼*3. 2-2-dimethyl-2-silapentane-5-sulfonate sodium salt (DSS) was used as the internal chemical shift reference.

	*δ***(ppm)**		*δ***(ppm)**
H2 (t)	3.82	C1	176.25
H3 (m)	2.17	C2	56.57
H4 (m)	2.58	C3	28.45
H6 (s)	9.54	C4	34.08
H8-z (d)	5.68	C5	176.54
H8-e (d)	5.74	C7	137.98
H10 (t)	8.56	C8	115.68
H11 (d)	4.00	C9	169.79
		C11	44.53
		C12	176.63
